# Plant community, ecosystem, and abiotic data from a global change experiment in mountain grasslands in Norway

**DOI:** 10.1038/s41597-025-06503-6

**Published:** 2026-01-07

**Authors:** Aud H. Halbritter, Joseph Gaudard, Helene Sandsten, Ieva Petrauskaite, Susanne Berthelsen, Gunnar Austrheim, Ingrid Dahle, Kari Klanderud, Linn Cecilie Krüger, Emma Little, Richard J. Telford, Vigdis Vandvik

**Affiliations:** 1 Department of Biological Sciences, University of Bergen, Bergen, Norway; 2 Bjerknes Centre for Climate Research, University of Bergen, Bergen, Norway; 3 Department of Ecology and Genetics, Uppsala University, Uppsala, Sweden; 4 Department of Natural History, NTNU University Museum, Norwegian University of Science and Technology, Trondheim, Norway; 5 Faculty of Environmental Sciences and Natural Resource Management, Norwegian University of Life Sciences, Ås, Norway; 6 Department of Civil Engineering and Geosciences, Delft Technical University, Delft, Netherlands

**Keywords:** Climate-change ecology, Biodiversity, Community ecology, Ecosystem ecology

## Abstract

Multiple global change drivers including land-use and climate change, and pollution threaten alpine biodiversity and ecosystem functions. Experimental approaches can be used to disentangle the single and interactive effects of these drivers. Across three sites along an elevational gradient (469–1290 m a.s.l.) in south-western Norway, we exposed plant communities to warmer climate, nitrogen fertilization, and grazing, as well as simulated grazing by clipping, in a split-plot design. After three years of treatment, we recorded data on vegetation, ecosystem functioning, and microclimate in 160 experimental and control plots. This database consists of records of the following datasets: aboveground standing biomass (3,417 records), aboveground plant productivity (2,071), reflectance (1,769), vascular plant community composition (8,954 records covering 95 taxa), belowground productivity and traits (796), soil characteristics (193), soil nutrient (1,132), ecosystem CO_2_ fluxes (2447), soil ecosystem CO_2_ respiration (64), and microclimate (30,751,264). The data can be combined with long-term climate and plant functional traits collected within the study region.

## Background & Summary

Mountains harbour a large proportion of the world’s biodiversity and also provide other important ecosystem functions and services including water, soil, and nutrient retention and regulation, carbon sequestration and storage, grazing areas for wildlife and domestic herbivores, and cultural and recreational services^1–3^. Alpine ecosystems are characterized to be temperature limited and nutrient availability is low. These conditions make ecological processes such as productivity, decomposition and respiration slow and thus alpine areas are potentially important carbon sinks^4,5^. Mountains are now impacted by multiple global change drivers^6^ threatening alpine biodiversity and ecosystem functioning and services^4,7^.

Anthropogenic global change drivers, such as land-use change, climate change, and pollution have a large impact on alpine ecosystems, causing extinctions, altering species composition, range dynamics, interactions, and ecosystem functions and services^6^. The climate in many mountains is locally warming at a faster rate than the global average^8^. Atmospheric nitrogen deposition has increased substantially in the last decades as a result of human activity^9^, even in remote alpine areas in south-western Norway^10^. Alpine ecosystems in the fjord landscapes of Norway have been managed for centuries as transhumance-based grazing systems (“utmarks-beite, seterbruk”)^11,12^. However, the land-use in many alpine grasslands has decreased and summer farms are being abandoned^13,14^. Global change impacts, in particular warming and nitrogen addition, on alpine ecosystems have been studied extensively^9,15,16^. These global change drivers do however not occur alone, but act simultaneously and can also interact^17,18^. Interactive effects of these drivers can be additive (i.e. the sum of the single effects) or multiplicative (i.e. greater or smaller than the single effects), which makes it difficult to predict their outcome^17–19^. Experiments combining multiple global change drivers are important to understand the underlying mechanisms of how these drivers affect biodiversity and ecosystem functioning^20–26^ and by replicating experiments along environmental gradients, the context dependency of such responses can be studied^27–30^.

**Fig. 1:**
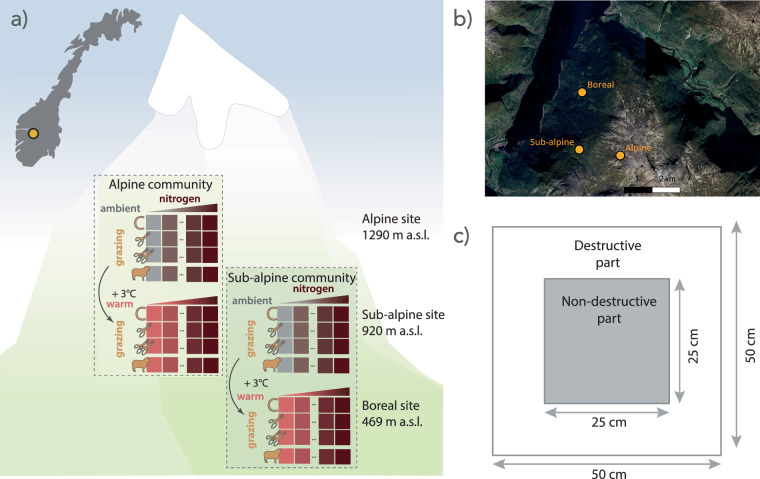
Experimental setup, location, and plot design. (**a**) Replicated experimental setup at the sub-alpine and alpine plant community along an elevational gradient with three sites. Inset shows map of Norway and location of experiment. The experiment includes a split-plot design of warming by transplanting (2 levels), nitrogen addition (8 levels) and grazing and clipping (4 levels). The treatments are ambient (grey), warming (pink), nitrogen addition (colour gradient), ungrazed (C), intermediate (one scissor) and intensive (two scissors) clipping, and natural grazing outside the grazing exclosure (sheep). The arrows indicate the direction of transplant. (**b**) Map showing the location of the three sites: boreal, sub-alpine, and alpine sites. (**c**) Plot design with the outer area for destructive sampling and inner non-destructive area for long-term monitoring. The global change treatments (warming, nitrogen addition, clipping and grazing) were applied on the whole 50 × 50 cm plot.

The ThreeD Global Change Experiment in Vestland County in Norway aims to better understand how multiple global change drivers affect alpine vegetation, biodiversity and ecosystem functioning. This experiment is a split-plot design, where we exposed grassland vegetation to three single global change drivers and their interactions, namely warmer climate, atmospheric nitrogen addition, and grazing or clipping (Fig. 1a). The experiment was replicated in an alpine and sub-alpine grassland community, representing a temperature and productivity gradient. Plant communities were exposed to a warmer climate by transplanting whole communities to lower elevation, to increased nitrogen deposition by fertilising, and to natural grazing by domestic and wild animals as well as clipping biomass at two different intensities. We applied a large range of nitrogen levels (0–150 kg N ha^−1^ y^−1^; see Methods) to simulate nitrogen addition close to and well beyond the current atmospheric nitrogen deposition (2–3 kg N ha^−1^ y^−1^) in the mountains in south-western Norway^10^ and the critical load (c. 5–10 kg N ha^−1^ y^−1^) for change in species composition in alpine grasslands^31^. Our understanding of environmentally extreme conditions is limited, and such simulations can provide a mechanistic understanding of thresholds for community change^32^. We used natural grazing and simulated grazing by clipping to disentangle the effect of biomass removal and other effects of grazing (i.e. fertilisation, trampling and selective grazing).

In this paper, we provide a detailed description of the site selection, the experimental design, data collection and also describe each dataset in detail. We collected 14 different datasets including (i) elevation and coordinates, (ii) slope, aspect, and soil depth, (iii) aboveground standing biomass, (iv) aboveground productivity and biomass consumption, (v) reflectance, (vi) belowground productivity and root traits, (vii) vascular plant community composition (cover), (viii) vascular plant community composition (presence), (ix) vegetation structure, (x) soil characteristics, (xi) soil nutrients, (xii) decomposition, (xiii) ecosystem CO_2_ fluxes and soil ecosystem CO_2_ respiration, and (xiv) microclimate (Table 1).

**Table 1:** Description and location of the datasets in the ThreeD global change experiment.

Dataset number	Response variable	Number of observations	Number of taxa	Number of plots	Treatments	Temporal range
i	Elevation and coordinates	3	—	—	—	2019
ii	Slope, aspect, and soil depth	480	—	160	all	2019
iii	Aboveground standing biomass	3,417	—	120	all	2020–2022
iv	Aboveground productivity functional group Aboveground productivity species Aboveground productivity and biomass consumption	1,758 199 114	— 49 —	36 15 18	G G G	2019 2019 2022
v	Reflectance	1,769	—	160	all	2020, 2022
vi	Belowground productivity and root traits Root scans	796 159	— —	160 —	all	2021, 2022
vii	Vascular plant community composition (cover) Species list Photos per plot	8,954 132 640	98 98 —	160 — —	all — all	2019–2022 - 2019–2022
viii	Vascular plant community composition (presence)	420,718	98	160	all	2019–2022
ix	Vegetation structure	4,237	—	160	all	2019–2022
x	*Soil characteristics:* Soil texture Bulk density Soil pH	42 50 15	— — —	7 37, 13 15	— — —	2019 2019, 2020 2019
xi	*Soil nutrients:* Soil organic matter Carbon and nitrogen stocks Available nutrients	278 648 206	— — —	30, 158 60, 26, 160 35	—, all —, —, all W, N (0, 5, 150 kg N ha^−1^ y^−1^), C	2019, 2021 2019, 2020, 2022 2021
xii	Decomposition	300	—	160	all	2021, 2022
xiii	Ecosystem CO_2_ fluxes Soil ecosystem CO_2_ respiration	447 2,000 64	— —	12 160 16	W all W	2020 2021 2021
xiv	Microclimate	7,687,816	—	75	W, N (0, 0.5, 1, 10, 100, 150 kg N ha^−1^ y^−1^), C	2019–2022

This table shows the dataset number, response variable(s), number of observations, number of taxa, number of plots, treatments, and temporal range of the data. The abbreviations in the treatments are W = warming, N = nitrogen addition, G = grazing, and C = clipping. The raw data are available on OSF^36^, the final published data are available on Zenodo^37^, and the code for extracting and cleaning raw data is available on GitHub, versioned on Zenodo^38^.

## Methods

### Data management and workflow

Data collection, management and workflows follow community approved standards^33–35^, including FAIR and Open Science data management and reproducible workflows. Our data are provided in data repositories. The raw data is available on the Open Science Framework (OSF)^36^ and the final clean data are available on Zenodo^37^. We process the data as minimally as possible and as much as necessary by fixing errors and typos, removing obvious erroneous data, flagging potentially biassed observations, and streamlining for full integration of all the datasets. All data cleaning and curation is done code based and fully transparent provided on GitHub versioned on Zenodo^38^. The availability of the raw data and processing code allows users to adapt the data according to their needs. For more details see the sections on Data Validation and Usage Notes below.

### Research site selection and characteristics

#### Site selection

The study was conducted at three semi-natural grassland sites distributed along an elevational gradient spanning 469–1290 m a.s.l. in Aurland, Vestland County in Norway (Fig. 1a,b). The sites cover three major bioclimatic zones in the study region, namely boreal (Vikesland, 469 m a.s.l., 60.88019 °N, 7.16990 °E), sub-alpine (Joasete, 920 m a.s.l., 60.86183 °N, 7.16800 °E), and alpine (Liahovden, 1290 m a.s.l., 60.85994 °N, 7.19504 °E). They differ in elevation by c. 400 m, corresponding to a difference of about 3 °C in summer temperature between the sites (mean of four warmest months; modelled climate data from the period of 2008–2021 by the Norwegian Meteorological Institute, met.no). While the sites differ in climate, they were selected to minimize variability in grazing history, vegetation type and structure, bedrock, slope, and exposure. Land use in the study system is free-range grazing and also includes natural grazers. Goats, sheep and deer are the dominant grazers at the boral and sub-alpine site and sheep and reindeer at the alpine site.

#### Site characteristics and vegetation

Air temperature during the growing season (May - September) decreased with elevation (boreal: 12.36 ± 0.17 °C, sub-alpine 9.12 ± 0.19 °C, alpine: 5.89 ± 0.20 °C, from *in situ* measurements during the study period between May - September 2019–2022). Annual precipitation is similar at the boreal (1292 mm) and sub-alpine site (1256 mm) and approximately 800 mm higher at the alpine site (2089 mm; modeled climate data from the period of 2008–2021 by Norwegian Meteorological Institute, met.no). All sites are located on calcareous bedrock and support a high diversity of grassland plant species. They are forb-rich, semi-natural grasslands where the boreal and sub-alpine site correspond to slightly calcareous semi-natural grassland (TK01-14) and the alpine site corresponds to calcareous alpine grasslands (TA03-06) according to the "Natur i Norge" (NiN) system for systematisation of natural diversity in Norway ^39^. The alpine site is dominated by *Silene acaulis, Thalictrum alpinum, Alchemilla alpina, Leontodon autumnalis, Antennaria dioica, Carex capillaris*, and *Festuca rubra*. The sub-alpine site is located close to a summer farm, and the vegetation is dominated by species adapted to grazing: *Achillea millefolium, Agrostis capillaris, Poa pratensis, Ranunculus repens*, and *Rumex acetosa*. The boreal site is located close to a farm, and the vegetation is dominated by *Potentilla erecta, Knautia arvensis, Agrostis capillaris, Rumex acetosa, Festuca ovina*, and *Poa pratense*. Annual plant productivity is similar at the boreal and sub-alpine site (10.20 ± 0.81 g m^−2^ y^−1^, 10.59 ± 1.1 g m^−2^ y^−1^, respectively) and about four times lower at the alpine site (2.56 ± 0.24 g m^−2^ y^−1^). Grazing pressure is 3–5 times higher in the sub-alpine site (1.48 ± 1.5 g m^2^ y^−1^, 1) which is located close to a summer farm, compared to the boreal (0.55 ± 0.48 g m^2^ y^−1^) and alpine site (0.28 ± 0.25 g m^2^ y^−1^).

### Experimental design

The experiment was replicated twice, at the alpine and the sub-alpine site (Fig. 1a). Within each of the sites, we set up a split-plot design, where each block received a different nitrogen addition treatment and within each block, each plot received a combination of warming and grazing/clipping treatments. Note that we used the terminology block and plot for the two levels. The design includes two factorial variables for warming (2 levels, randomized for plots within block for a total of 40 plots/treatment level) and grazing/clipping (4 levels, randomized for plots within each warming treatment and block for a total of 20 plots/treatment level), and one quantitative variable for nitrogen addition (8 levels; gradient approach applied across blocks). Note that there are three replicates for no nitrogen addition to facilitate analysis of warming and grazing effects independent of fertilization.

At the start of the growing season in 2019, ten experimental blocks with eight 50 × 50 cm plots were established at each site (n = 160, Fig. 1a). The sites were fenced in early spring 2020, before the animals were released to the outfield, to exclude the domestic and natural grazers from the experimental plots. Two plots per block were located outside the fence to allow for natural grazing by large herbivores. The plots outside the fence were < 10 m from the adjacent block inside the fence, but not too close to be affected by the fence. Each plot was permanently marked with four aluminium pipes in each corner for resampling. The plots had an inner 25 × 25 cm area marked with the same method, and used for continuous, non-destructive measurements, while the outer area was used for destructive sampling and harvest (Fig. 1c). The global change treatments (see below) were applied on the whole 50 × 50 cm plot. The upslope left corner tubes of the inner and outer area were marked with a colour-coded waterproof tape to ensure the same orientation of the plots for all measurements.

The three treatments were randomly allocated in two steps. First, each of the ten blocks was randomly assigned a *nitrogen addition treatment*, to avoid nitrogen contamination between the plots within the blocks. If a block had a nitrogen treatment that was > 2 levels higher than the block downslope from the block, the nitrogen treatments were switched. This procedure was repeated until no nitrogen contamination was possible from upslope blocks. The following eight nitrogen treatments were assigned: 0, 0.5, 1, 5, 10, 50, 100, 150 kg N ha^−1^ y^−1^, where three blocks were controls and received no nitrogen addition. Nitrogen was added to each plot using slow dissolving fertilizer pellets (YaraBela OPTI-NS 27-0-0 (4S)). We used oxidised nitrogen (NO_x_) formed mainly by combustion processes^40^, which are the main sources of atmospheric nitrogen deposition in remote regions (i.e., away from intensive agriculture and other sources of reduced nitrogen such as ammonia). The nitrogen was added once at the start and once in the middle of the growing season from 2020–2022, except in 2020 at the lowest site, where the whole dose of fertilizer was applied by accident at the start of the season.

Second, the *warming, grazing, and clipping treatments* were randomised within each block, with the exception of the two naturally grazed plots, that were randomly assigned outside the fenced area. At the end of August in 2019, half of the plots of entire plant communities were excavated to below the rooting depth and transplanted to lower elevation, simulating an approximately 3.0 °C warmer growing season temperature. The aim was to transplant an intact rooting system, resulting in a variation in rooting depth (range: 5–25 cm), because the soil is heterogeneous and rocky. Soil depth was 3.3 cm deeper at the sub-alpine site compared to the alpine site. The control plots were not excavated. All available evidence indicates that whole community transplants have little impact on grassland ecosystems, specifically community composition and plant performance^27,41^. The transplanting was conducted within 24 hours. The upslope left-hand corner of each transplanted community was marked with a toothpick to allow placing the transplanted plot in a similar position relative to the slope and block orientation at the destination site. If necessary, loose soil was carefully removed from the underside of the turf, or local soil was added to the gap or around the edges to ensure the soil surface was in plane with the surrounding vegetation.

The four *grazing or clipping treatments* consisted of control (inside grazing exclosure with no grazing or clipping), intermediate clipping, intensive clipping or natural grazing (outside grazing exclosure). The clipping treatment was used to simulate clipping intensity and to disentangle the effect of biomass removal from other effects of grazing (i.e. selective grazing, fertilization, and trampling). The intermediate and intensive clipped plots were cut two or four times during each growing season, respectively, about 2–3 cm above the ground. The plots located outside the fence were naturally grazed by domestic and wild animals (see above). In 2020, the intensive clipped plots at the alpine site were only cut three times because the growing season was short due to late snowmelt. An additional cut at the end of the growing season would not have yielded any biomass. In 2021 and 2022, we did not cut *Carex* species before the species composition was analyzed in early August (first and second cut), because some sedges are difficult to identify without leaf tips.

#### Plot naming design

Each plot was given an *origin* (orig) and a *destination* (dest) siteID, blockID, and plotID referring to a specific location at the origin or destination site. For the control plots that were not moved, the origin and destination IDs are identical. Each plot also received a unique turfID referring to the turf that was moved from origin to destination. The turfID consists of the origPlotID, treatments and destPlotID. For the origPlotID each plot was given a number from 1 to 160, starting at the alpine site and continuing the counting at the sub-alpine site. The treatment is a combination of letters and numbers related to the warming, nitrogen addition and grazing or clipping treatment (for more details see Table 2). The destPlotID is a number between 1 and 200 related to the location the plot was transplanted to. The turfs transplanted to the boreal site were given destPlotIDs 161–200.

**Table 2:** Data dictionary for linking variables that occur in all datasets.

Variable name	Description	Variable type	Variable range or levels	Units	How measured
origSiteID	Unique site ID of origin site	categorical	Joasete–Liahovden		defined
origBlockID	Unique origin block ID as number 1 to 10	numeric	1–10		defined
origPlotID	Unique numeric origin plot ID for geographic location of the turf	numeric	1–160		defined
destSiteID	Unique site ID of destination site	categorical	Joasete–Vikesland		defined
destPlotID	Unique destination block ID as number 1 to 10	numeric	2–200		defined
destBlockID	Unique numeric destination plot ID for geographic location of the turf	numeric	1–10		defined
turfID	Unique ID of vegetation turf as origin plotID, warming, nitrogen and grazing treatment and destination plotID	categorical	1 WN1M 84–99 WN5C 170		defined
warming	Warming treatment as W for warming or A for ambient	categorical	A–W		defined
grazing	Grazing treatment as C for control, I for intensive, M for medium clipping, and N for natural grazing	categorical	C–N		defined
Nlevel	Nitrogen level treatment as numeric value from 1–10	numeric	1–10		defined
Namount_kg_ha_y	Amount of nitrogen added between 0 and 150 kg per ha and year	numeric	0–150	kg ha^−1^ y^−1^	defined

Data dictionary with variable name, descriptions, variable type, variable range or levels, units and how measured.

### Species identification, taxonomy, and flora

All vascular plant species were identified to the species level in the field, with nomenclature following Lid and Lid^42^. Exceptions are juvenile plants and sterile specimens that are not possible to identify without reproductive parts, and where flowers are either too rare or individuals too short-lived for comparisons of the position of individuals within the plots over years to be used to ascertain identifications (For example, *Alchemilla* spp. excluding *A. alpina*). Species identifications were confirmed by comparing records over time as described below. All unidentified specimens (see section Technical Validation for exact numbers of unidentified species) are included and flagged in the dataset, as described below. The full species names are provided in the taxon table on OSF^36^.

### Additional datasets

**Fig. 2:**
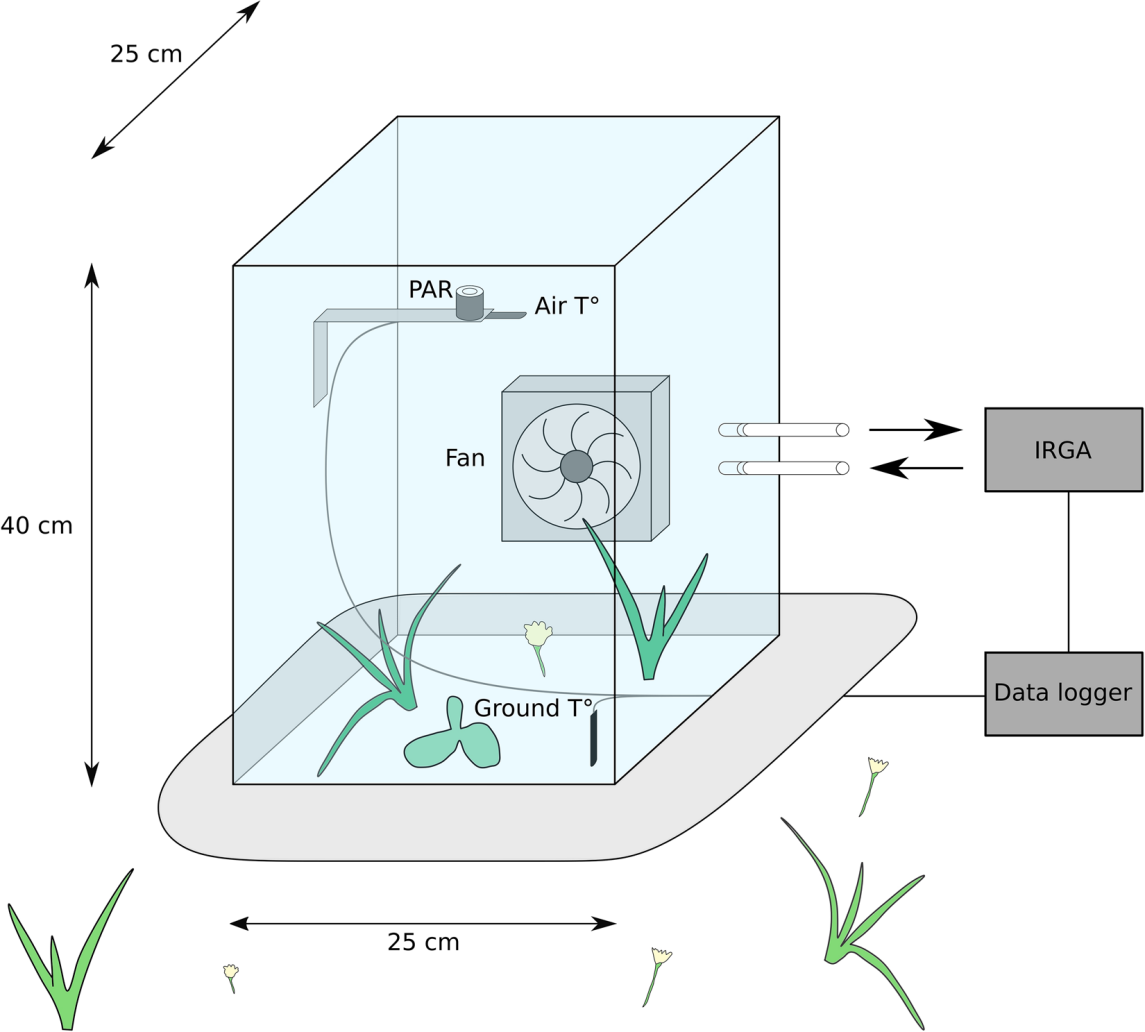
Close-loop chamber system for ecosystem gas flux measurements. The chamber (25 × 25 × 40 cm) is connected to an Infrared gas analyser (IRGA) and has sensors for air temperature, soil temperature (2 cm depth), and photosynthetically active radiation (PAR). A fan ensures good mixing of the air inside the chamber and a skirt weighed down with a heavy chain makes it airtight on the ground.

The sixth Plant Functional Trait Course used the ThreeD Global Change Experiment in 2022, and additional data was collected at the site and plot level^43^. The data collected during this campaign are plant and leaf functional traits, leaf assimilation-temperature (AT) responses, leaf handheld hyperspectral readings, leaf temperature measurements, diurnal ecosystem CO_2_ fluxes, and landscape-scale airborne multispectral (10-band) and RGB cm-resolution imagery from airborne sensors. These data are described in detail^43^ and available on OSF^44^. The data are fully compatible with the data described in this paper (Fig. 2).

The ThreeD Global Change Experiment shares the boreal site with the Vestland Climate grid (VCG)^27,45^ and is part of the same study system. There are additional data from previous experiments in the VCG that are relevant for the data presented here, in particular, the vascular plant community composition and climate data^27,45^. These data are also available on OSF^46^.

The code to access and download relevant datasets from the Plant Functional Trait Course 6 and VCG data repositories is provided on the Plant Functional Trait Course 6 GitHuB repository^47^.

### Dataset collection methods

#### Dataset (i): Elevation and coordinates

Elevation, latitude, and longitude of each site were recorded at the start of the experiment in 2019, using a Global Positioning System device (Garmin Montana 610). These data are available as elevation, and coordinates per site (Table 1).

#### Dataset (ii): Slope, aspect, and soil depth

In 2019, we measured slope and aspect for each plot using a clinometer and a compass. For measuring soil depth, we stuck a metal pole, perpendicular to the slope as far down into the soil as possible at each corner of every plot and measured the length of the pole that went into the soil. These data are available as slope, aspect, and soil depth per plot (Table 1).

#### Datasets (iii) – Aboveground standing biomass

Aboveground standing biomass was harvested in several rounds between 2020–2022 for the clipped plots (see clipping treatment above). At peak growing season in 2022, aboveground biomass was also harvested for the ungrazed plots (controls). The biomass in the clipping treatments reflects the biomass consumed by grazers. The biomass collected at peak growing season in the ungrazed plots represents the remaining standing biomass.

The aboveground biomass was cut 2–3 cm above the ground on the whole 50 × 50 cm plot as it is a global change treatment (Fig. 1c). For the clipping treatment, biomass was cut 2 × (intermediate) or 4 × (intensive) during the growing season (see clipping treatment above). The biomass for each plot was sorted to plant functional groups, namely graminoids with Cyperaceae sorted separately, forbs (including herbaceous forbs, pteridophytes), legumes, shrubs (including dwarf-shrubs, and small individuals of trees and shrubs), bryophytes, lichen and litter (including dead non-attached biomass), dried at 65 °C for 72 h and weighed. These data are available as biomass per plot and sampling date (Table 1).

#### Datasets (iv) – Aboveground plant productivity and biomass consumption

To measure aboveground plant productivity and biomass consumption by grazers along the elevational gradient we used permanent and movable grazing exclosures (i.e. cages that exclude large herbivores)^48^. The summed biomass in the control plots represents plant productivity, while the difference of the biomass in the grazing exclosure and the control plots represents the biomass consumed by the grazers. Applying permanent and movable grazing exclosures repeatedly over the growing season thus enables an estimate of annual plant productivity and biomass consumption. The intervals between moving the cages have to be chosen carefully depending on the natural grazing intensity in the system, because it can otherwise over- or underestimate plant productivity.

At the start of the growing season in 2019, we installed twelve 30 × 30 cm cages (1 cm mesh size) and associated control plots at each site. Three cages and control plots were left at the same location for the whole growing season (permanent), while the other cages and control plots were moved after each harvest, approximately every four weeks. The biomass from each plot was then harvested 2–3 cm above the ground. The biomass was sorted to functional groups (forbs, graminoids, and shrubs), dried at 65 °C for 72 h and weighed. In July, the biomass was also sorted to species level, dried at 65 °C for 72 h and weighed. Note that in addition, soil moisture and pH were collected for each plot and can be retrieved from the raw data. For methodology see associated readme files.

At the start of the growing season in 2022 we installed three 30 × 30 cm cages (1 cm mesh size) and associated control plots at each site. Approximately every two weeks (15.7 ± 1.25 days), representing an intermediate grazing density, the biomass from each plot was harvested 2–3 cm above the ground. The cage and control plots were moved after each harvest to a new, ungrazed location for the next sampling interval, because we were interested in biomass consumption and not the regrowth of the vegetation. This procedure was repeated six times in the boreal and sub-alpine site and five times at the alpine site during the growing season. The harvested biomass was dried at 65 °C for 72 h and weighed. These data are available as biomass per plot and sampling date (Table 1).

#### Dataset (v): Reflectance

Red and infrared light reflectance of the plants is an estimate of Normalized Difference Vegetation Index (NDVI) and were taken in several rounds for each plot during the field season in 2020 and for one round in 2022 using a Trimble Greenseeker Handheld Crop Sensor (Trimble Inc., Sunnydale, CA, USA). In 2020 NDVI measurements were taken 4–6 times in each plot, corresponding to the ecosystem CO_2_ measurements done (see dataset xiii). In 2022 only one measurement was done at the alpine site during peak growing season.

The sensor measures an elliptical shape, and the light source and receiver was pointed at the middle of the plot. We made two measurements per plot perpendicular to each other to account for the elliptical shape. The measurements covered the 50 × 50 cm area of each plot (Fig. 1c). The measure was taken 60 cm above the plot and parallel to the ground when possible. These data are available as reflectance per plot per sampling date (Table 1).

#### Dataset (vi): Belowground productivity and root traits

To measure belowground productivity, i.e. fine root growth and belowground traits, we installed two root ingrowth cores (RIC)^49^ in the destructive sampling area of each plot (Fig. 1b) at the start of the growing season (mid-June) in 2021. The RICs were made of plastic netting tubes with a diameter of 3.5 cm and a mesh size of 2 mm. They were 10 cm long, but because of the varying soil-depth in the plots some were shorter. To install the RICs, a soil core was extracted from each plot, and the soil was sieved with 2 mm mesh size removing roots and stones. A RIC of the size of the soil core was filled with the extracted soil, and in a few cases soil from the surrounding area was added. We did not add the stones back into the RIC, because the soils were generally very stony but variable across plots, and we wanted similar and optimal growing conditions for all root ingrowth cores. Note that this approach changes the bulk density of the soil (see Usage Notes).

One core per plot was collected after one growing season in September (105 ± 0.6 days) to assess g*rowing season belowground productivity*. The second core was collected after one full year in May–June 2022 (363 ± 0.2 days) to assess *annual belowground productivity*. To extract the cores, we cut around the edges of the RIC to prevent the roots from being pulled out and twisted the core around before pulling it out of the soil. The samples were transported within 2–3 days to the lab and stored in a freezer at −22 °C until further processing.

The root samples were taken out of the freezer to thaw a few hours before processing. The length of each RIC was measured and noted down to calculate the soil volume. The excess roots were carefully cut and the soil on the outside was removed. The RIC was placed in water, above a 500 μm sieve and the roots were carefully washed in multiple rounds until only roots were left. We only spent a maximum of 60 minutes on each sample to standardise the processing time. Note that we did not sort between dead and alive roots and the root productivity contains both.

The first batch of samples (*growing season productivity*) were dried at 65 °C for 72 hours and weighed, and we only assessed root productivity. For the second batch (*annual productivity*), the excess water was removed by gently tapping and moving the roots in a tray, before measuring the wet weight. The roots were stored up to 48 h in a folded paper soaked in distilled water in a refrigerator before measuring root traits.

We used a flatbed scanner (Epson perfection v800/v850 3.93 (32-32)) to scan the roots and the *WinRhizo* software to measure root traits. We used the default settings, except for *resolution* 600 dpi, turned off *speed priority*, and *Regent Positioning System* to 15 × 20 cm. The roots from each sample were placed in a plexiglas tray filled with distilled water and carefully spread as much as possible before scanning the roots. The sample name and the root ingrowth core volume in m^3^ was added before analysing and saving the image. The roots were then placed in paper bags, dried at 65 °C for 72 hours and weighted.

##### Data processing

We calculated the following root traits: root productivity, specific root length, root tissue density and root dry matter content using the following equations:

1 $${Root\; productivity}(g\,{{cm}}^{-3}{{time}}^{-1})={Root\; dry\; mass}/{soil\; volume}$$

2 $${Specific\; root\; length}(m\,{g}^{-1})={Root\; length}/{Root\; dry\; mass}$$

3 $${Root\; tissue\; density}(g\,{{cm}}^{-3})={Root\; dry\; mass}/{Wet\; root\; volume}$$

4 $${Root\; dry\; matter\; content}({mg}{g}^{-1})={Root\; dry\; mass}/{Root\; wet\; mass}$$

Note that time in Eq. 1 refers to either the growing season or a whole year and is indicated with the variable period (i.e. growing season or annual).

These data are available as root productivity and traits per plot and sampling period (Table 1). The root scans are also available (Table 1).

#### Datasets (vii, viii, ix): Vascular plant community composition and vegetation structure

Vascular plant species community composition was recorded in each plot at peak growing season in 2019 (before starting the treatments) in 2021, and 2022 (after treatments). In 2020 we recorded community composition only in the ambient and warmed plots. To facilitate species identification, we chose a time in between two clipping events (*grazing treatment*), to ensure that most species were fully grown and the leaves were not clipped.

Species composition was recorded on the inner 25 × 25 cm non-destructive area of each plot used for long-term monitoring (Fig. 1c), using a subplot overlay dividing each plot into 25 subplots. We first recorded the presence of each vascular plant species in each subplot and additionally, if the individual was dominant (D; covered more than 50% of the sub-plot), fertile (F; bud, flower, seeds were present), juvenile (J; not yet adult), or a seedling (S; cotyledons still attached; S). For the entire 25 × 25 cm plot, we then visually estimated the percentage cover of each vascular plant species to the nearest 1%, and total cover of bryophytes, lichen, litter, bare ground, rocks and feces. Note that the total coverage in each plot can exceed 100, due to layering of the vegetation. At four fixed points in the plot, average vegetation height and moss layer depth was measured using a ruler. A photo of each plot was taken each year.

For species identification we followed the nomenclature from Lid and Lid^42^. Each species of vascular plant was classified by functional type (graminoid, forb, legume, woody) based on information given in Lid and Lid^42^. Species that could not be identified to species level without flowers or seeds, were indicated with *sp* (e.g. *Carex*, *Luzula* and *Antennaria* sp).

The vascular plant vegetation data is available as percentage cover per species per plot per sampling date as well as a separate list with all species and functional groups (vii), presence and life history status (fertility, juvenile, seedling) per species, subplot and sampling date (viii), and vegetation structure and vegetation height and moss depth in cm per plot per sampling date (ix; Table 1). In addition, a photo of each plot and year during peak growing season is available (Table 1).

#### Dataset (x, xi): Soil characteristics and nutrients

##### Soil sampling and processing

Soil samples were taken to measure soil characteristics (*dataset x*) and soil nutrients (*dataset xi*), including soil texture (sand, silt and clay content), bulk density, pore water content, pH, soil organic matter, carbon and nitrogen stocks, and available nutrients. We used the same soil samples for the soil characteristics and nutrient measurements and therefore first describe the soil sampling in the field and the initial processing steps in the lab in this section. For further details on the processing and analysis see the description of each of the dataset below.

##### Sampling time and number

In peak growing season 2019, two soil samples per site were collected from each block (n = 60), but outside of the plots. The soil samples were 5 cm in diameter and were taken as deep as possible, on average 12.8 ± 0.6 cm deep (range 4.0 – 24.0 cm). Each sample was divided into the upper organic layer, and if possible, the lower mineral soil. At the end of the growing season 2020, we collected five random soil samples per site in between the blocks (n = 26). These samples are linked to two or three blocks and therefore more useful at the site level. The soil samples were 5 cm in diameter and only the upper 4 cm of soil was collected. If there was a mineral layer, the soil sample was divided into organic and mineral layers. At the end of the growing season in 2021 and 2022, one soil sample from the destructive part of each plot was taken (Fig. 1c; 2021: n = 158; 2022: n = 160). The samples were 2.5 cm in diameter and were taken as deep as possible (mean depth: 7.2 ± 0.2 cm).

##### Processing

All soil samples were frozen at −22 °C until further processing. The block level samples from 2019 and 2020 were weighed before drying to estimate pore water content (see below). A sub-sample of the soil used for loss on ignition was air dried first (see below). All soil samples were dried at 60 °C for 48 h and sieved with a 2 mm sieve to remove stones and roots and the remaining dried soil was weighed. For the samples in 2019 and 2020 we also weighed the roots and stones in each sample to estimate bulk density. Each soil sample was well mixed to homogenise before further processing.

##### Soil characteristics (x)

Soil characteristic measurements include soil texture, bulk density, and soil pH with one sample from each block and site in 2019 (n = 60) and 5 samples per site in 2020 (n = 26). For details on the soil sampling and processing see above.

##### Soil texture

Soil texture, the sand, silt and clay content, was measured using the particle size analysis^50^. Sand, silt and clay have different particle sizes and will set in water at different rates. The organic and mineral layer of three random soil samples per site from 2019 (n = 7) were well mixed. A jar was filled with one third of soil and two thirds of water plus some dishwashing powder. The solution was mixed thoroughly for several seconds. Then the jars were left on the bench and after distinct time intervals the layers that had set in the water, were marked on the jar. The sand layer was marked after one minute, the silt layer after two hours, and the clay layer after 48 hours. The height of each layer was used to calculate the proportion of sand, silt and clay in the soil sample. Soil texture can be used to define the soil type using the soil texture triangle.

##### Bulk density

Bulk density was estimated from 37 samples in 2019 and 13 samples in 2020. For each sample we weighed the dried soil, and stones. Dry bulk density was then calculated using the following equations:

5 $${Bulk\; density}(g\,{{cm}}^{-1})=({dry\; weight\; core}(g)-{stone\; weight}(g))/({core\; volume}({{cm}}^{3})-{stone\; volume}({{cm}}^{3}))$$

6 $${Stone\; density}( \sim 2.65g{{cm}}^{-3})={stone\; mass}(g)/{stone\; volume}({{cm}}^{3})$$

##### Soil pH

The pH was measured in a soil water solution^51,52^, using five random samples per site from 2019 (n = 15). From each sample, 10 g of fresh field-moist soil was weighed into a 50 ml plastic pH beaker. 50 ml of deionised water was added and the suspension was stirred thoroughly. After 30 minutes with occasional stirring, the soil pH was measured electrometrically using the calibrated pH metre. The pH metre was calibrated in a buffer solution of pH 4, 7 and 9 after every fifth sample.

These data are available as sand, silt and clay content, and bulk density, and pH per site, sampling depth and date (Table 1).

##### Soil nutrients (xi)

Soil nutrient measurements include soil organic matter, carbon and nitrogen stocks, as well as available nutrients. For details on the soil sampling and processing see above.

##### Soil organic matter

Soil organic matter was measured using the loss on ignition method^53^. We used one sample per block in 2019 (n = 30) and one sample per plot in 2021 (n = 158). An amount of circa 10 g of air-dried and homogenised soil was weighed into ceramic crucibles and weighed. The weight of each ceramic crucible was noted. The soil samples were dried at 105 °C for 16 hours and weighed again (soil and crucible). The samples were then burned at 550 °C for six hours and weighed (soil and crucible). Finally, the samples were burned at 950 °C and a final weight (soil and crucible) was recorded. The weight of the crucible was then subtracted from each measurement.

We calculated the soil water content, organic matter and carbonate content using the following equations:

7 $${\rm{Soil\; water\; content}}({{\rm{weight}}}_{{\rm{air\; dried}}}-{\rm{dry}}{{\rm{weight}}}_{105^\circ {\rm{C}}})/{{\rm{weight}}}_{{\rm{air\; dried}}}$$

8 $${\rm{Soil\; organic\; matter}}({{\rm{weight}}}_{105^\circ {\rm{C}}}-{\rm{dry}}{{\rm{weight}}}_{550^\circ {\rm{C}}})/{{\rm{weight}}}_{105^\circ {\rm{C}}}$$

9 $${\rm{Soil\; carbonate\; content}}({{\rm{weight}}}_{550^\circ {\rm{C}}}-{\rm{dry}}{{\rm{weight}}}_{950^\circ {\rm{C}}})/{{\rm{weight}}}_{105^\circ {\rm{C}}}$$

##### Carbon and Nitrogen content

The carbon and nitrogen content was measured from one sample per block and site in 2019 (n = 60), five samples per site in 2020 (n = 26), and one sample per plot and site in 2022 (n = 160). The dried soil samples were homogenized and a subsample was sent to external labs to measure carbon and nitrogen content by dry combustion^54,55^. The samples from 2019 and 2020 were analysed by the LabTek at the Norwegian University for Life Sciences in Ås, and the samples from 2022 were analysed by Bionér, Bø, Norway.

##### Available soil nutrients

Plant Root Simulator (PRS^TM^; Western Ag Innovations, Inc., Saskatoon, Canada) simulate roots and give an estimate the amount of available nutrients in the soil. In 2021 we installed three PRS probes per plot (n = 35) with ambient and warmed climate, crossed with 0, 10 and 150 kg N ha^−1^ y^−1^, and three clipping treatments (control, intermediate and intensive clipped). The probes were installed for 35 days during peak growing season (July - August), after which they were retrieved, brought back to the lab, washed with deionized water and shipped to the Western Ag Innovations, Saskatoon, Canada for analysis. The analysis includes cations and anions of N, P, K, S, Ca, Mg, Al, Fe, Mn, Cu, Zn, B, Pb, and Cd.

Measurements of P and Zn at the alpine site and Cd, K, Mn, and S at the sub-alpine and alpine site were below the detection limit and therefore excluded from the dataset.

These data are available as soil organic matter, carbonate content, water content, total soil carbon, and total soil nitrogen content, and available soil nutrients per plot or site, sampling depth and date (Table 1).

#### Dataset (xii): Decomposition

To measure decomposition we used the Tea bag Index protocol^56^. We used green tea (Lipton green tea: EAN 87 22700 05552 5) and rooibos tea (Lipton rooibos: EAN 87 11327 5143 48), because they decompose at different rates which allows to calculate the rate of decomposition (*k*) and the stabilisation factor (*S*). Two bags of green and rooibos tea each were weighed and buried at the start of the growing season (late May - mid June) 2021 in each plot (n = 320 green and n = 320 rooibos teabags). The soil was slit open with a knife and the tea bags were carefully buried at up to 10 cm depth. We recorded the burial depth of the tea bags, because the soil was very shallow and stony in some plots, preventing the burial of all the tea bags at the same depth.

We collected one pair of tea bags at the end of the growing season in late September 2021 (*growing season decomposition*) and the other pair of tea bags at the start of the growing season in May - June in 2022 (*annual decomposition*). The first batch were thus buried for one growing season (106 ± 0.86 days, range 94–119 days) and the second batch for a whole year (363 ± 0.23 days, range 359–378 days). The tea bags were transported to the lab, cleaned of soil outside the teabag, dried at 65 °C for 48 hours and weighed.

Note that we did not use the correct Lipton rooibos tea suggested by the Tea bag Index protocol^56^ and comparing these data to other TBI data might not be meaningful (see Usage notes).

These data are available as mass loss, decomposition rate and stabilisation factor per plot and date (Table 1).

#### Dataset (xiii): Ecosystem CO_2_ fluxes and soil CO_2_ respiration

##### Ecosystem CO2 fluxes

Ecosystem CO_2_ fluxes were measured during four campaigns spread over the whole growing season in 2020 and 2021. In 2020 all ambient and warmed plots without nitrogen addition, clipping, or grazing were measured (n = 12). In 2021 fluxes were measured on all plots (n = 160). In 2020, warming and control plots were measured three times during each campaign. In 2021 each plot was measured once during each campaign (n = 160).

Net Ecosystem Exchange (NEE) was measured using a transparent chamber, allowing photosynthetic CO_2_ uptake and respiratory CO_2_ release from the ecosystem. Ecosystem Respiration (ER) was measured by covering the chamber with a light-excluding, dark cloth. We estimated Gross Primary Production (GPP) from these measurements.

##### Field measurements

CO_2_ fluxes were measured using a closed loop chamber system (Fig. 2), following the method by Jasoni *et al*.^57^. A tarp was attached at the bottom of the chamber and weighed down with a heavy chain to seal the chamber to the ground during measurements to prevent air mixing between the chamber and the outside. The plexiglas chamber (25 × 25 × 40 cm) was connected with an infrared gas analyzer (IRGA; Li-840, LI-COR Biosciences, Lincoln, NE, USA) for measuring CO_2_ concentration. The chamber was equipped with a fan for optimizing air circulation and the IRGA was connected with flexible plastic tubing and an air pump to ensure a flow of 1 L min^−1^ through the IRGA. A filter at the start of the incoming air tube prevented water droplets and small particles from entering the IRGA. The chamber was also equipped to record air temperature with an iButton (iButtonLink Technology, Whitewater, USA) in 2020 and a thermocouple (Pt1000, Delta-T) in 2021, soil temperature (2 cm depth) with a thermocouple (Pt1000, Delta-T) in 2021 only, and a photosynthetically active radiation (PAR) sensor (LI-190/R Quantum sensor, Li-COR).

In 2020, PAR was logged at 15 seconds intervals (Li-COR Li4000) and air temperature at 10 seconds intervals. In 2021, PAR, air and soil temperature were logged at 10 seconds intervals (Squirrel Data Logger 2010 Series, Grant Instruments).

We used the following protocol for measuring CO_2_ concentration in each plot: first the chamber was kept 1 m above the ground for 1 min to air the chamber and tubes and prevent the accumulation of CO_2_. The starting time for each measurement was recorded manually. Then the chamber was placed over the plot, sealed with the chain and CO_2_ was measured continuously for 120 seconds in 2020 and 180 seconds in 2021, first under light conditions and then the whole procedure was repeated under dark conditions.

##### Soil CO_2_ respiration

Soil CO_2_ respiration was measured four times during the growing season in 2021, corresponding with the ecosystem CO_2_ flux measurement. The measurements were done in one ambient and one warmed plot at each site (n = 16).

Soil CO_2_ respiration was measured using the chamber method on permanently installed collars. In November 2020, collars were installed in the destructive area of one ambient and one warmed plot at each site. They were 10 cm in diameter and 20 cm long, entering ca. 7 cm deep into the soil. The depth of the collars varied for each chamber due to difference in soil depth and rockyness and each measurement was corrected for by the chamber volume. The collars were installed eight months before respiration measurements for the soil to recover from the disturbance. The vegetation inside the collars was regularly removed to avoid measuring plant respiration.

The collar was sealed with an air-tight plastic lid, and tubes were connected to an infrared gas analyser (IRGA, LI-84A, LI-COR). For each plot, the setup was aired for 1 minute, to prevent the accumulation of CO_2_ in the chamber and tubes. Then the lid was put on the collar closing the chamber and CO_2_ was measured every second for 180 seconds.

##### Data processing and calculations

The flux data were processed with the fluxible R package^58^, using the exponential model ‘exp_zhao18,^59^ in the flux_fitting function. For the 2020 data the entire length of the measurements was used, while for the 2021 dataset the last 30 seconds of each measurement were cut to improve the quality of the fit.

These data are available as fluxes per plot and sampling date (Table 1).

#### Datasets (xiv) – Microclimate data

Microclimate data was recorded continuously (every 15 min), between August 2019 and September 2022, using Tomst TM4 loggers with accuracy of ± 0.5 °C^60^. Air temperature was measured 15 cm above the ground and at ground level (0 cm), and soil temperature and soil moisture were measured 8 cm belowground. The loggers were placed in one corner of the destructive area of the plot, in all warming and clipping treatments inside the fence (control, medium and intensive) and at 0 (in two out of three blocks), 0.5, 1, 10, 100 and 150 kg N ha^−1^ y^−1^ (total number of loggers: n = 75). The 0.5 kg N ha^−1^ y^−1^ treatment only had climate loggers at the alpine site, because this block was located further apart from the others at that site. At the sub-alpine site, the 0.5 kg N ha^−1^ y^−1^ treatment had climate loggers in 2019 and partly in 2020 but were then moved to the 10 kg N ha^−1^ y^−1^ treatment at the alpine and sub-alpine sites. Loggers that failed to record data were replaced. These data are available as temperature or percentage soil moisture per plot and sampling date (Table 1).

## Data Records

**Fig. 3:**
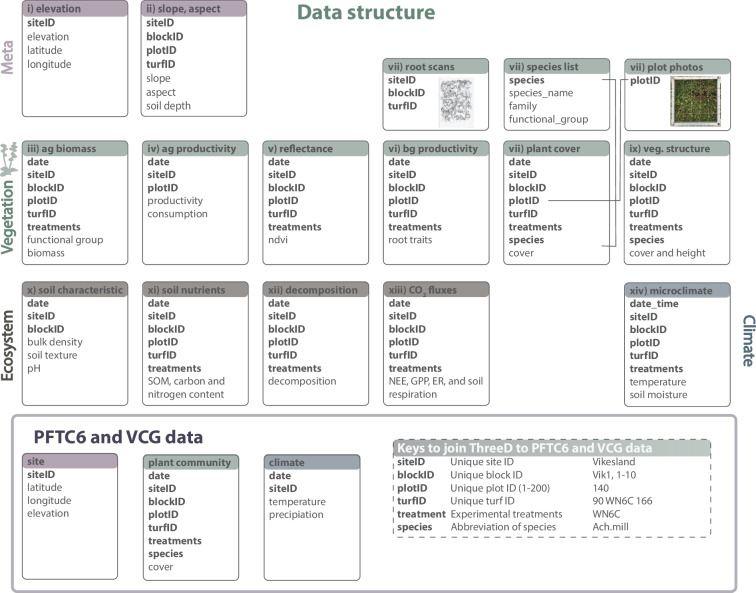
Data structure for the ThreeD global change experiment. The boxes represent the dataset i to xiv (corresponding to Table 1) with the names of each dataset shown in the coloured area. Note that the dataset names are shortened and abbreviated in this figure because of limited space (ag = aboveground, bg = belowground, veg = vegetation). The main variables of each dataset are shown below and for the full list of variables for each dataset see Tables 2–16. The variables in bold indicate keys that can be used to join the different datasets (Table 2). The line linking the variable species indicates one example for such a link. These keys can also be used to link to data from the Plant Functional Trait Course 6 (PFTC6) and the Vestland Climate Grid (VCG) in Vestland County in Norway and are explained in the box at the bottom. Three example datasets with their keys are shown. Abbreviations in variable names stand for: SOM = soil organic matter, veg. = vegetation, nee = net ecosystem exchange, gpp = gross primary production, and ER = ecosystem respiration.

Here we give an overview of the data repository, and the organization and structure of the 14 datasets (Fig. 3, Table 1).

### Data organization and structure

The raw data files are available on OSF^36^ and are stored in folders using the dataset number and response variable used in Table 1. Each filename also starts with a dataset number corresponding to Table 1. All raw data files are indicated with “raw” and have an associated readme file. The readme file or a tab in the dataset called readme explains each variable in the data files.

The final data files are available on Zenodo^37^. The file names follow the same structure: nr_Three-D_clean_variable_year(s).csv, where nr refers to the dataset number in Table 1, the variable corresponds to the response variable in Table 1. The code to clean and manage the data files on OSF is available in the ThreeD GitHub repository with a versioned copy archived in Zenodo^38^.

All datasets are structured similarly, sharing some common variables including year, date, (orig/dest)siteID, -blockID, -plotID, turfID, and treatments and specific variables that are unique to each dataset (Fig. 3). The shared variables can be used to link different datasets, for example to combine them for specific analysis (bold letters in Fig. 3). For example, the climate data can be joined by date, siteID, blockID, plotID, turfID and treatments to the biomass to test the relationship between soil temperature and biomass.

The variables from all datasets are explained in separate data dictionaries in the respective sections below (see Tables 3–16). All linking variables (e.g. siteID, blockID) are only explained once, in the data dictionary below (Table 2) to avoid repetition.

**Table 3:** Data dictionary for elevation and coordinates (dataset i).

Variable name	Description	Variable type	Variable range or levels	Units	How measured
elevation_m_asl	Elevation of site	numeric	469–1290	m a.s.l.	recorded
latitude_N	Latitude of site	numeric	60.86–60.88	degree N	recorded
longitude_E	Longitude of site	numeric	7.168–7.195	degree E	recorded
bioclimatic_zone	Bioclimatic zones of the sites including alpine, sub-alpine and boreal	categorical	alpine - sub-alpine		defined

Data dictionary with column descriptions for dataset i – site level elevation and coordinates. Note that any linking variable is not included in this data dictionary and is explained in Table 2.

**Table 4:** Data dictionary for slope, aspect and soil depth (dataset ii).

Variable name	Description	Variable type	Variable range or levels	Units	How measured
year	Year of the sampling	numeric	2019–2019	2019	recorded
date_slope	Date of sampling slope and aspect	categorical	2019-07-04–2019-07-17	yyyy-mm-dd	recorded
slope	Slope of the plot	numeric	0.3–280	degree	measured
aspect	Aspect of the plot	numeric	2–380	degree	measured
date_depth	Date of sampling soil depth	categorical	2019-07-17–2019-06-24	yyyy-mm-dd	recorded
soil_depth_cm	Soil depth measurement of the plot	numeric	4.625–36.1	cm	measured
remark	Comment on measurement, recording or observation.	categorical			recorded

Data dictionary with column descriptions for dataset ii – plot level slope, aspect and soil depth. Note that any linking variable is not included in this data dictionary and is explained in Table 2.

**Table 5:** Data dictionary for above ground biomass (dataset iii).

Variable name	Description	Variable type	Variable range or levels	Units	How measured
year	Year of the sampling	numeric	2020–2022	2019	recorded
date	Date of sampling	date_time	2010-09-10–2022-09-01	yyyy-mm-dd	recorded
cut	Number of cuts for the intermediate and intensive clipping treatments	numeric	1–4		recorded
fun_group	Functional group including forbs, graminoids, Cyperaceae, legumes, bryophytes, and litter	categorical	bryophytes–shrub		defined
biomass	Amount of biomass removed per plot	numeric	0–86.54	g	measured
unit	Unit for biomass is gram	categorical		g	recorded
area_cm2	Plot size in cm2	numeric	223–2500	cm^2^	recorded
collector	Data collector	categorical			recorded
remark	Comment on measurement, recording or observation	categorical			recorded

Data dictionary with column descriptions for dataset i – biomass data from 2020–2022.

**Table 6:** Data dictionary for aboveground plant productivity (dataset iv).

Variable name	Description	Variable type	Variable range or levels	Units	How measured
date	Date of sampling	date	2019-05-28 - 2019-09-20	yyyy-mm-dd	recorded
date_in	Date when cage/control plot was set up	date_time	2022-05-31–2022-08-18	yyyy-mm-dd	recorded
date_out	Date when cage/control plot was harvested	date_time	2022-05-31–2022-09-02	yyyy-mm-dd	recorded
campaign	Number of times measured during the growing season	numeric	1–3		recorded
duration	Time between biomass harvests.	numeric	8–25	days	calculated
treatment	Grazed control plots (Control) and ungrazed cages (Cage). The summed biomass in the caged plots is annual plant productivity and the difference in biomass between cage and control is the consumed biomass.	categorical	Cage–Control		recorded
plot_nr	Number of replicates.	numeric	1–3		recorded
type	Type of measurement including permanent (i.e. same location) and temporary (i.e. change location after each harvest) placement of the plots.	categorical	permanent - temporary		recorded
replicate	Replicate measurement	numeric	1–4		recorded
area_cm2	Plot size in cm^2^	numeric	900–900	cm^2^	recorded
functional_group	Functional group including forbs, graminoids, and shrubs	categorical	bryophytes - shrubs		recorded
species	Latin genus and species name	categorical	Achillea millefolium - Viola palustris		identified
productivity	Amount of biomass produced between each harvest.	numeric	0.001–0.043		measured
remark	Comment on measurement, recording or observation	categorical			recorded

Data dictionary with column descriptions for dataset iv – productivity data from 2022. Note that any linking variable is not included in this data dictionary and is explained in Table 2.

**Table 7:** Data dictionary for reflectance data (dataset v).

Variable name	Description	Variable type	Variable range or levels	Units	How measured
year	Year of the sampling	numeric	2020–2022	year	recorded
date	Date of sampling	date	2020-06-26–2022-07-27	yyyy-mm-dd	recorded
timing	Timing of measurement in relation to cutting treatment	categorical	after 1. cut–before treatment		recorded
replicate	Replicate measurement of same turf	numeric	1–2		recorded
ndvi	Normalized Difference Vegetation Index measured per plot	numeric	0.29–0.96	percentage	measured
remark	Comment on measurement, recording or observation	categorical	flat light–shadow		recorded
flux_campaign	Campaign connected to carbon flux measurements	numeric	2–4		recorded

Data dictionary with column descriptions for dataset v – reflectance data from 2020 and 2022. Note that any linking variable is not included in this data dictionary and is explained in Table 2.

**Table 8:** Data dictionary for belowground root productivity and trait data (dataset vi).

Variable name	Description	Variable type	Variable range or levels	Units	How measured
year	Year of the sampling	numeric	2021–2022	2019	recorded
sampleID	Soil sample ID	categorical	100AN5M100–9AN6M9		defined
days_buried	Duration of root growth in days			days	calculated
period	Period for root productivity measurement: growing season in 2021 or annual in 2022	categorical	annual - growing season		defined
variable	Belowground productivity (g cm^−3^ y^−1^) and root traits, including specific root length (m g^−1^), root tissue density (g cm^−3^), and root dry matter content (mg g^−1^)	categorical	root_dry_matter_content - specific_root_length		defined
value	Value for belowground productivity or root trait	numeric	0 - 366.168	g cm^−3^ y^−1^, m g^−1^, g cm^−3^, mg g^−1^,	measured
volume_cm3	Root ingrowth core volume	numeric	36.56–101.984	cm3	recorded
burial_date	Date of incubation of root ingrowth core	date	2021-05-30 - 2021-06-21	yyyy-mm-dd	recorded
recover_date	Date of recovery of root ingrowth core	date	2021-09-20 - 2022-06-29	yyyy-mm-dd	recorded

Data dictionary with column descriptions for dataset v – reflectance data from 2020 and 2022. Note that any linking variable is not included in this data dictionary and is explained in Table 2.

**Table 9:** Data dictionary for the community composition (dataset vii).

Variable name	Description	Variable type	Variable range or levels	Units	How measured
year	Year of the sampling	numeric	2019–2022	2019	recorded
date	Date of sampling	date	2019-07-02 - 2022-08-12	yyyy-mm-dd	recorded
species	Latin genus and species name	categorical	*Achillea millefolium* - *Viola palustris*		identified
cover	Percent cover of a species per plot	numeric	1–80	percentage	recorded
recorder	Name of person that recorded the data	categorical			recorded
scribe	Name of scribe	categorical			recorded
remark	Comment on measurement, recording or observation	categorical			recorded
file	Name of file in the raw data	categorical			recorded

Data dictionary with column descriptions for dataset vii – plant community cover data from 2019–2022.

**Table 10:** Data dictionary for the subplot community composition (dataset viii).

Variable name	Description	Variable type	Variable range or levels	Units	How measured
year	Year of the sampling	numeric	2019 - 2022	2019	recorded
date	Date of sampling	date	2019-07-02 - 2022-08-12	yyyy-mm-dd	recorded
subplot	Location of subplot within the plot	categorical	1–9		defined
species	Latin genus and species name	categorical	*Achillea millefolium* - *Viola palustris*		identified
variable	Variable including presence, fertility, dominant ( > 50% of cover), juvenile or seedling	categorical	dominant - seedling		defined
value	Presence or absence indicated with 1 for presence and 0 for absence	logical	0–1		recorded
recorder	Name of person that recorded the data	categorical			recorded
remark	Comment on measurement, recording or observation	categorical			recorded

Data dictionary with column descriptions for dataset viii – subplot community composition data from 2020–2022.

**Table 11:** Data dictionary for the vegetation structure (dataset ix).

Variable name	Description	Variable type	Variable range or levels	Units	How measured
year	Year of the sampling	numeric	2019–2022	2019	recorded
date	Date of sampling	date	2019-07-02 - 2022-08-12	yyyy-mm-dd	recorded
variable	Variables including cover (the functional group cover of each plot; %), height (vegetation; cm), and depth (bryophyte; cm).	categorical	cover - sum_cover		defined
functional_group	The functional groups include bryophytes, litter, vascular plants, lichen, faeces, bare soil, bare rock and wool; The variable sumofcover is the sum of cover of all species including the layering of vegetation and can exceed 100%; vegetation refers to the height of the vegetation and bryophyte to the moss depth.	categorical	bare rock - wool		defined
value	Value for cover, sumofcover, height, and depth.	numeric	0–177	percentage or cm	recorded
recorder	Name of person that recorded the data	categorical			recorded

Data dictionary with column descriptions for dataset ix – vegetation structure data from 2019–2022.

**Table 12:** Data dictionary for soil structure (dataset x).

Variable name	Description	Variable type	Variable range or levels	Units	How measured
year	Year of the sampling	numeric	2019–2020	2019	recorded
date	Date of sampling	date_time	2019-07-16 - 2020-10-16	yyyy-mm-dd	recorded
layer	Layer at which soil sample was taken: top or bottom 5 cm	categorical	Bottom - Top		recorded
variable	Soil character variable including bulk density, pore water content, percentage sand, silt and clay, and pH	categorical	bulk_density_g_cm - silt_percent		defined
value	Soil character value	numeric	0.082–97.222	gcm^−3^, %	measured

Data dictionary with column descriptions for dataset x – soil structure data from 2019 and 2020.

**Table 13:** Data dictionary for soil nutrients (dataset xi).

Variable name	Description	Variable type	Variable range or levels	Units	How measured
year	Year of the sampling	numeric	2019–2022	2019	recorded
date	Date of sampling	date_time	2019-07-16 - 2019-07-22	yyyy-mm-dd	recorded
sample_ID	Unique ID of sample	numeric	1–160		defined
layer	Layer at which soil sample was taken: top or bottom 5 cm	categorical	Bottom - Top		recorded
variable	Soil nutrient variable including carbon and nitrogen content (%), soil organic matter (%), and the elements Al, B, Ca, Cd, Cu, Fe, K, Mg, Mn, NH^4-^N, NO^3-^N, P, Pb, S and Zn (grams/10 cm^2^/35 days)	categorical	Al - Zn		defined
value	Soil nutrient value	numeric	0.009 - 2513.14	%, micro grams/10 cm^2^/35 days	measured
duration	Duration of PRS probe in the soil	days	35	days	recorded
detection_limit	Detection limit of element	numeric	0.04 - 717.53		defined
burial_date	Burial date of PRS probe	date	2021-07-07 - 2021-07-08	yyyy-mm-dd	recorded
retrieval_date	Retrieval date of PRS probe	date	2021-08-11 - 2021-08-12	yyyy-mm-dd	recorded
soil_depth_cm	Soil depth of CN samples	numeric	3–11.5	cm	recorded
Notes	Comment on measurement, recording or observation	categorical			recorded

Data dictionary with column descriptions for dataset xi – soil nutrients data from 2019 and 2022.

**Table 14:** Data dictionary for decomposition (dataset xii).

Variable name	Description	Variable type	Variable range or levels	Units	How measured
year	Year of the sampling	numeric	2021–2022	2019	recorded
teabag_ID	Unique tea bag ID	numeric	1–340		defined
timing	Time of recovery after one growing season or a whole year	categorical	growing season - year		recorded
incubation_time	Incubation time of tea bags in days	numeric	94–378	days	recorded
k	Decomposition rate	numeric	0.002–0.095		calculated
S	Stabilization factor	numeric	0.125–0.838		calculated
fraction_remaining_green	Fraction of green tea remaining after burial period	numeric	0.263–0.864	%	calculated
fraction_remaining_red	Fraction of rooibos tea remaining after burial period	numeric	0.422–0.865	%	calculated
flag	Flagging problematic data	categorical			recorded

Data dictionary with column descriptions for dataset xii – decomposition data from 2021 and 2022.

**Table 15:** Data dictionary for ecosystem CO_2_ fluxes (dataset xiii).

Variable name	Description	Variable type	Variable range or levels	Units	How measured
date_time	Date and time of sampling or observation	date_time	2020-06-26 12:59:10 - 2021-09-10 12:55:10	yyyy-mm-dd_hh:mm:ss	recorded
type	Type of flux measurements (ER, NEE or GPP; numbers designate light response curves)	categorical	1 - NEE		defined
temp_soil_ave	Soil temperature 2 cm belowground inside the chamber during flux measurement	numeric	7.143 - 25.882	degree celsius	measured
comments	Comments on flux measurements	categorical	maybe loose tube - windy		recorded
f_quality_flag	Quality flags as provided by flux_quality	categorical	discard - zero		recorded
plot_area	Area of plot used for gas flux measurements	numeric	0.008 - 0.062	m^2^	recorded
f_temp_air_ave	Air temperature measured inside the flux chamber every 10 seconds and averaged	numeric	8.25 - 38.9	Kelvin	measured
chamber_vol	Volume used for gas flux measurements (chamber + tubing)	numeric	0.9 - 24.575	liter	recorded
PAR_ave	PAR value measured every 15 seconds during flux measurement and averaged	numeric	−0.005 - 2133.714	micromol/s/sqm	measured
flux_campaign	Campaign for the carbon flux measurements	numeric	1–4		defined
f_flux	CO_2_ flux (positive when emitting to atmosphere, negative when vegetation uptake)	numeric	−307.623 - 156.747	mmol/sqm/h	calculated
par_correction	if TRUE, NEE fluxes were standardized at PAR = 300 umol m^-2 s^-1 and ER fluxes at PAR = 0 umol m^-2 s^-1	logical	NA - TRUE		defined
replicate	Replicate measurement of same turf	numeric	1–3		defined

Data dictionary with column descriptions for dataset xiii – ecosystem CO_2_ flux data from 2020 and 2021, including soil respiration.

**Table 16:** Data dictionary for microclimate (dataset xiv).

Variable name	Description	Variable type	Variable range or levels	Units	How measured
date_time	Date and time of sampling or observation	date_time	2019-08-22 23:15:00 - 2022-09-02 05:45:00	yyyy-mm-dd_hh:mm:ss	recorded
air_temperature	Air temperature 15 cm above ground	numeric	−22.3125 - 47.0	°C	measured
ground_temperature	Ground temperature at ground level	numeric	−17.0 - 45.0	°C	measured
soil_temperature	Soil temperature 8 cm belowground	numeric	−9.875 - 43.375	°C	measured
soilmoisture	Volumetric soil moisture	numeric	−0.02589339 - 0.6201382	°C	measured
loggerID	Unique ID of logger	categorical	94194604 - 94201707		defined
shake	Shake values	numeric	202 - 202		recorded
error_flag	Flag for error of logger	numeric	0–33		recorded
remark	Comment on measurement, recording or observation	categorical			recorded

Data dictionary with column descriptions for dataset xv – microclimate data from 2019 to 2022.

### Dataset (i) Elevation and coordinates

This dataset contains elevation, latitude, and longitude for each site and has three observations. For an overview of the clean dataset see Table 3.

### Dataset (ii) Slope, aspect, and soil depth

This dataset contains slope, aspect and soil depth and has 480 observations. For an overview of the clean dataset see Table 4.

### Dataset (iii): Aboveground standing biomass

This dataset contains the aboveground standing biomass from the clipping treatments in 2020 to 2022 and from the control plots in 2022 (Table 5). For an overview of the clean dataset see Table 5.

### Dataset (iv): Aboveground plant productivity

This dataset contains the aboveground plant productivity from grazed control plots and ungrazed caged plots at all three sites in 2019 (at functional group and species level) and 2022 (at functional group level (Table 6) and has a total of 2,071 observations. For an overview over the clean dataset see Table 6.

### Dataset (v): Reflectance

The reflectance dataset contains a total of 1,769 observations (Table 7). For an overview over the clean dataset see Table 7.

### Dataset (vi) Belowground productivity and root traits

The belowground productivity (i.e. fine root productivity) and root traits dataset contains a total of 796 observations (Table 8). For an overview over the clean dataset see Table 8.

### Datasets (vii, viii): Vascular plant community composition

The vascular plant community composition dataset contains a total of 98 identified taxa and 8,954 observations of cover data at the plot level (Table 9). For an overview over the clean dataset see Table 9.

The subplot level vascular plant community composition dataset contains presence data of a total of 98 taxa and 420’718 observations (Table 10). For an overview over the clean dataset see Table 10.

### Dataset (ix): Vegetation structure

The vegetation structure dataset has 4’237 observations of functional group cover, sum of cover of all vascular species as well as vegetation height and bryophyte depth (Table 11). For an overview over the clean dataset see Table 11.

### Dataset (x): Soil structure

The soil structure dataset contains a total of 193 observations from 2019 and 2020 (Table 12). For an overview over the clean dataset see Table 12.

### Dataset (xi): Soil nutrients

This dataset contains a total of 1132 observations from 2019 to 2022 (Table 13). For an overview over the clean dataset see Table 13.

### Dataset (xii): Decomposition

The dataset contains a total of 300 observations, with 155 from one growing season in 2021 and 145 for a whole year from spring 2021 to spring 2022 (Table 14). For an overview over the clean dataset see Table 14.

### Dataset (xiii): Ecosystem CO_2_ fluxes

The 2020 CO_2_ fluxes dataset shows 447 measurements of ER, NEE and GPP (calculated from ER and NEE). The 2021 CO_2_ fluxes dataset shows 2064 measurements of ER, NEE and GPP (calculated from ER and NEE) and soil respiration. For an overview of the dataset see Table 15.

### Dataset (xiv): Microclimate

This dataset contains air, ground and soil temperature and soil moisture data from 80 loggers with a total of 7,687,816 observations, per plot and year (Table 16). For an overview over the clean dataset see Table 16.

## Technical Validation

### Experimental validation

This global change experiment was run between summer 2019 (before treatment) and 2022, and the treatments were applied for three years. Consistency in the leadership ensures reliability in the experiment and data collection. AHH led the fieldwork for the whole project, while many students were helping to conduct the work in the field and lab. Any errors and inconsistencies in the experiment, treatments and data collection were recorded and are available in the comments. These comments were also used for flagging data (also see Usage Notes).

The microclimate data shows that the warming treatment increased air, soil and ground temperature by approximately 3 °C, which was the intended amount of warming we wanted to achieve (*dataset xiv*). The data from the PRS probes show that available NH_4_^+^ and NO_3_^-^ increased exponentially with increasing nitrogen addition (*dataset xi*), suggesting that nitrogen availability indeed increased in the soil. The standing biomass data shows a decrease in the clipped plots, showing that the biomass removal treatments had the intended outcome (*dataset iii*). The standing biomass in the clipping treatments was lower compared to the unclipped control plots, suggesting that the clipping did not stimulate plant growth.

### Taxonomic validation

**Fig. 4:**
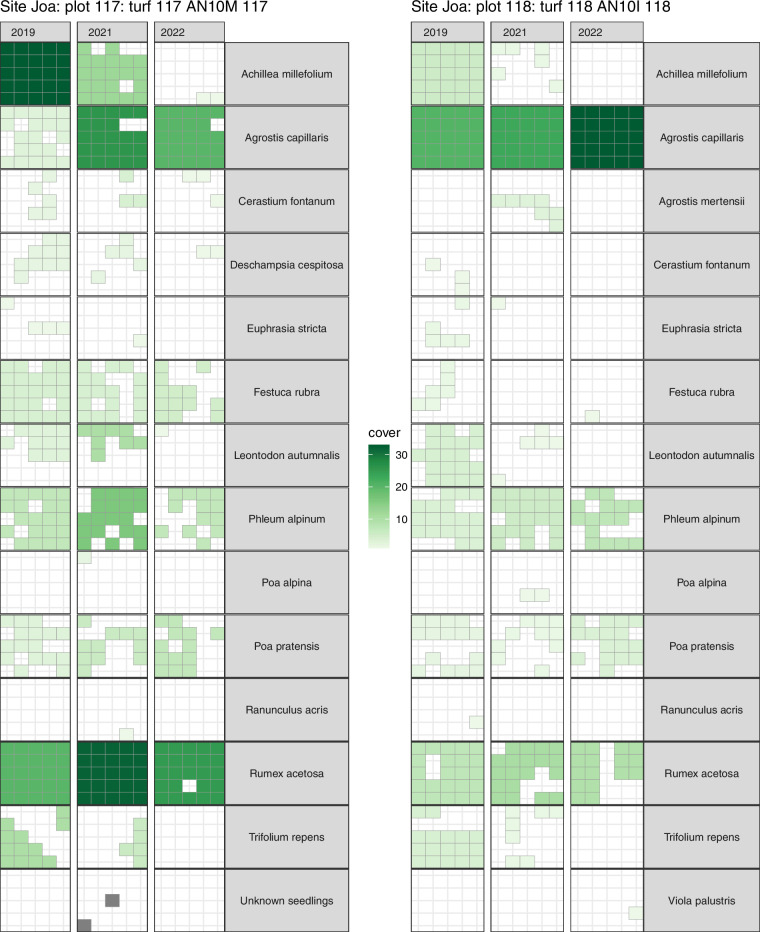
Graph produced by turfmapper showing the change in species presence and cover over time. Two example plots showing sub-plot presence and cover for each species in a time series from 2019, 2021, and 2022. Each green square shows the presence of a species in the subplot per time step. The colour indicates the percentage cover of a species at the plot level. These maps can be used to illustrate change in species composition and abundance over time and to identify misidentifications of species, wrong estimations of cover and to help correcting these issues in the data. Note that any correction in the data should be carefully considered and justified, in particular in the plots with global change treatments.

The plant community composition data (*datasets vii-ix*) was collected by a total of eight researchers. AHH always led the data collection and three of the researchers were always part of the data collection ensuring consistency in the data collection and reducing the risk of observer bias. Sterile graminoids and juvenile species can be difficult to identify, and rare species can be missed during one observation and affect the community composition data. We took several measures to correct such errors. The data was checked visually by plotting species cover over time using the vegetation time series visualisation R package turfmapper^61^. We compared the species and their cover in these time-series (Fig. 4) to identify missing or unidentified species, to detect misidentifications (i.e. species identity changes between years), and correct missing cover or cover estimates that were clearly too high or low. In addition to the time series, we used photographs of each plot and year which were also useful to check species IDs and make some corrections.

In the community composition cover dataset (*dataset vii*), ten forbs and three graminoid taxa were identified to the genus level (542 observations, 6.1%). In addition, four graminoids, six forbs, and one unknown plant were not identified and are labelled as unknown graminoid, unknown herb or unknown juvenile (0.15%). The presence data (*dataset viii*) contains 9.5% of observations with fertile plants and 1.2% of the observations are from dominant species covering >50% of the plot. In addition, 1% of observations are from juvenile plants and 0.3% from seedlings.

We provide a species list with all identified and unidentified taxa on OSF^36^.

### Ecosystem C flux validation

The ecosystem CO_2_ fluxes datasets were processed and validated using the fluxible R package^58^. In particular, we used the functions flux_quality and flux_plot for the validation step. Fluxes for which the model fit was not satisfactory but showed a change of gas concentration in the chamber over time were discarded, whereas those with an unsatisfactory fit and no change were replaced by zero. The quality of the model fit was assessed using the root mean square error (RMSE; a bad fit was defined as a RMSE value >25) and the b parameter of the exponential model (bad fit when outside the range [−1−1]). The occurrence of change of concentration over time was assessed with the correlation coefficient between concentration and time (no change in concentration over time was defined as a value within the range [−0.5−0.5]). For the 2021 dataset, the last 30 seconds of the measurements were cut, as a large proportion of measurements showed noise in that interval.

After visual inspection with the flux_plot function, 6 measurements in the 2020 dataset and 8 measurements in the 2021 dataset were manually attributed different quality flags because their quality had been wrongly assessed by the flux_quality function (Table 17). GPP fluxes have been calculated in a further step, and are therefore not included in the quality assessment. They inherited the quality flags of NEE fluxes and were not calculated if one of the paired fluxes (NEE or ER) was discarded.

**Table 17:** Quality summary of ecosystem gas flux measurements.

Quality flag	2020 dataset	2021 dataset
ok	284	1333
zero	15	80
force_discard	4	4
discard	1	4
force_lm	1	2
force_zero	1	0
start_error	0	0
no_data	0	0
force_ok	0	1
no_slope	0	0

Number of quality flags for 2020 and 2021 datasets after quality control with flux_quality and visual assessment with flux_plot. The flags with the “force_” prefix have been manually attributed following the visual assessment. GPP fluxes are not included as they have been calculated after the quality assessment.

## Usage Notes

### Experimental treatments

Transplanting to lower elevation increases the mean air, ground and soil temperature of the transplanted communities by ca. 3 °C. Like most experimental approaches, transplanting has other side effects that users should be aware of, including an increase in growing season length and change in soil moisture (i.e. it is drier at the sub-alpine and boreal site). The datasets presented here include a range of environmental measurements that can be used to account for these side effects.

Aboveground biomass at the highest nitrogen level (150 kg N ha^−1^ y^−1^) decreased considerably. This effect on biomass could indicate that the high level of nitrogen fertilization affected the soil pH resulting in acid soil conditions. Users should consider excluding the highest level of nitrogen addition from analysis.

### Transplant effect

The ambient plots differ from the warmed plots as they were not excavated and locally transplanted at the same location. Local transplants are a way to control for the transplanting effect. We did not include a local transplant control due to workload and because previously we had not found a transplant effect on the plant community composition^27,41^. Users should be aware that the transplant effect was not controlled for and could affect for example belowground biomass, soil characteristics, or soil nutrients.

### Variable soil depth

The transplanted communities were excavated below the rooting depth. The soil depth of the transplanted and control communities naturally varied, due to heterogenous and rocky soil. Soil depth is slightly deeper at the sub-alpine site compared to the alpine site (see methods), but we found no treatment bias in the soil depth of the communities. Note that the variation in soil depth could have an effect on the plant community and ecosystem. This variation can however be accounted for by including soil depth in analyses (dataset ii).

### Aboveground biomass (dataset iii)

The biomass in the clipping treatments was cut in all years and represents the biomass consumed by grazers. The biomass in the ungrazed control plots was only harvested in the last year of the experiment (2022) and represents standing biomass. The remaining biomass in the naturally grazed plots was not harvested. Standing biomass for all plots and treatments can be estimated from the cover and vegetation height in datasets vii and ix.

### Aboveground plant productivity and biomass consumption (dataset iv)

This dataset contains plant productivity per plot and sampling period. To estimate biomass consumption by the grazers, the difference between the biomass in the grazing exclosure and the control plots can be calculated.

### Belowground productivity (dataset vi)

Root productivity was estimated using root ingrowth cores (RIC). Roots and stones were removed when filling the soil back into the RICs. This was done because the soils were generally very stony and variable and we wanted to achieve similar growing conditions in all plots. Note that this approach changes the bulk density and generates optimal growing conditions for root growth. We measured bulk density at the block and site level (*dataset x*), which can be used to correct for root productivity.

### Vascular plant community composition (datasets vii, viii, ix)

We took a picture for each plot and year, which can help to visualize the effects of the different treatments. In 2019 four photos are missing (turfID: 88 AN1N 88, 90 WN6C 166, 106 WN3I 174, 135 WN4N 188) and in 2020 and 2021 one is missing in each year (2020 turfID: 71 WN9 151, 2021 turfID: 50 AN4M 50). The percentage of faeces in the naturally grazed plots (outside the fence) was not consistently recorded. The photos taken from each plot can be used to estimate this at peak growing season. However, it will not give an estimate for the whole growing season.

*Anthoxantum odoratum* and *A. alpinum* both occur in the study sites and can be difficult to distinguish when sterile. A genetic study on these two *Anthoxantum* species in the study area has shown that plants growing at the boreal site are *A. odoratum*, while the species growing close to the sub-alpine site (Høgsete) and generally at alpine sites are *A. alpinum*^62^. All Anthoxantum individuals in this study originate from the alpine and sub-alpine site and were therefore assigned *A. alpinum*. The individuals that over the course of the experiment colonize the plots at the boreal site can either be *A. alpinum* from the seedbank (originating from the sub-alpine site) or *A. odoratum* colonizing from the boreal site and unless fertile are difficult to distinguish. We have therefore decided to assign all *Anthoxantum* individuals at the boreal site also to be *A. alpinum*. We leave it up to the user to decide how to deal with this uncertainty.

## Data Availability

The raw data are available on OSF^36^, the final published data are available on Zenodo^37^.

## Code availability

The code for downloading the raw data from the data repository, data transformation, analyses and producing this manuscript are available on GitHub, versioned on Zenodo^38^. The whole workflow is (hope) fully reproducible by using a target pipeline^63^ and a reproducible environment via the R package renv^64^.
